# Interpreting Molecular Monitoring Results and International Standardization in Chronic Myeloid Leukemia

**DOI:** 10.6004/jadpro.2012.3.3.3

**Published:** 2012-05-01

**Authors:** Stephanie Bauer, Edie Romvari

**Affiliations:** From Bone Marrow Transplant Division in the School of Medicine, Washington University, St. Louis, Missouri

## Abstract

Important advances in the understanding of the molecular basis of chronic myeloid leukemia have resulted in the development of new therapies and changed the paradigm for managing this myeloproliferative disease. Translocation of chromosomes 9 and 22 (known as the Philadelphia chromosome) results in a fusion *BCR-ABL* gene that produces a dysregulated BCR-ABL tyrosine kinase protein and triggers events leading to malignant transformation. The tyrosine kinase inhibitors imatinib, nilotinib, and dasatinib block the BCR-ABL protein and prevent activation of the transformation pathways. Molecular monitoring, the most sensitive approach currently available to assess treatment response, measures *BCR-ABL* messenger RNA levels and serves as a surrogate marker of disease. Further, molecular responses are predictive of patient outcomes. It is important for advanced practitioners to become familiar with the technology and interpretation of molecular monitoring results as well as efforts to standardize this type of testing so they can educate their patients and aid their understanding of test results. Undetectable *BCR-ABL* levels can bring feelings of relief, whereas an increasing level can lead to anxiety. Advanced practitioners, therefore, are an important resource for interpreting results for patients, answering questions, alleviating concerns, and encouraging continued adherence to treatment.

Chronic myeloid leukemia (CML) affects 1 to 2 people per 100,000 annually, with an estimated 5,000 patients diagnosed in the United States each year (Altekruse et al., 2009). The underlying cause of CML is a translocation between chromosomes 9 and 22 that results in an abnormal chromosome known as the Philadelphia (Ph) chromosome. The Ph chromosome is composed of pieces from chromosome 9 and 22 that have fused, giving rise to the leukemogenic BCR-ABL gene. The *BCR-ABL* gene expresses the *BCR-ABL* tyrosine kinase (TK) protein (Figure 1), which has unregulated activity and triggers a cascade of events culminating in malignant transformation (Mauro & Druker, 2001). The ultimate goal of CML treatment is to eliminate the BCR-ABL protein and prevent transformation to later phases of disease, which are inherently more difficult to treat than the initial chronic phase.

**Figure 1:**
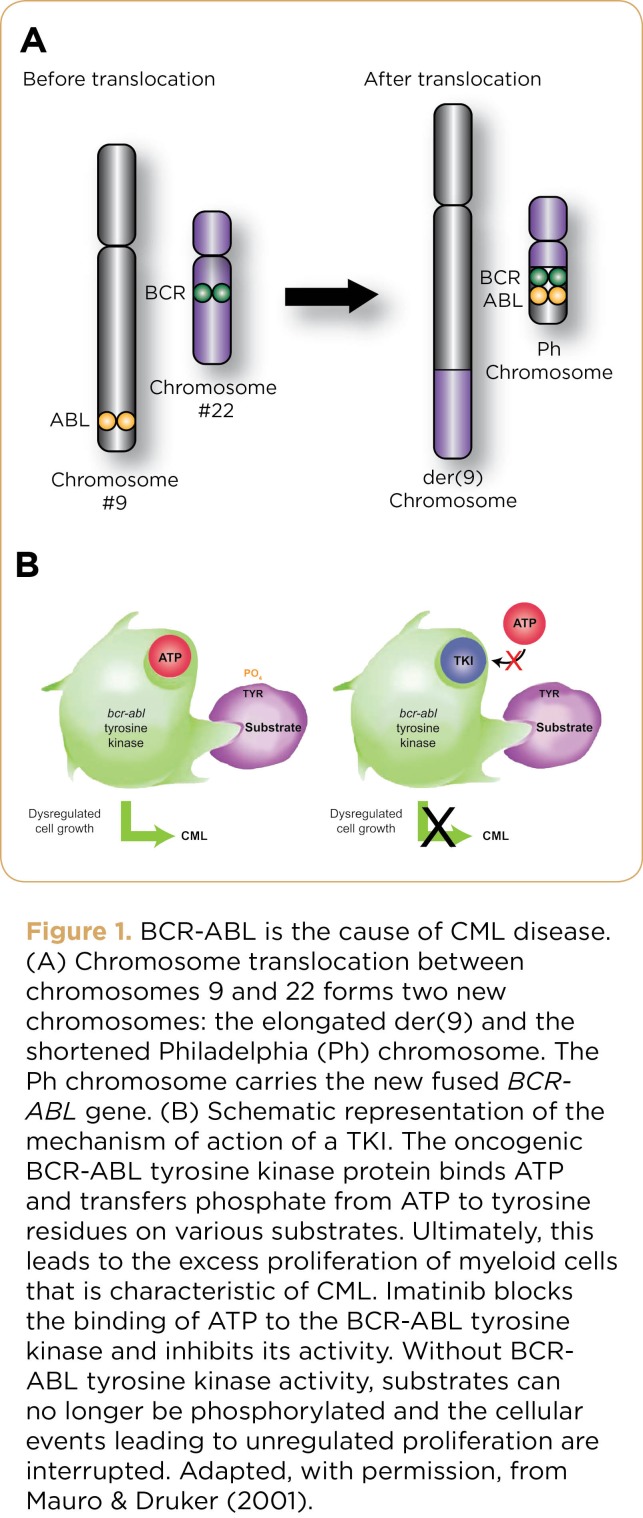
Figure 1. BCR-ABL is the cause of CML disease. (A) Chromosome translocation between chromosomes 9 and 22 forms two new chromosomes: the elongated der(9) and the shortened Philadelphia (Ph) chromosome. The Ph chromosome carries the new fused BCRABL gene. (B) Schematic representation of the mechanism of action of a TKI. The oncogenic BCR-ABL tyrosine kinase protein binds ATP and transfers phosphate from ATP to tyrosine residues on various substrates. Ultimately, this leads to the excess proliferation of myeloid cells that is characteristic of CML. Imatinib blocks the binding of ATP to the BCR-ABL tyrosine kinase and inhibits its activity. Without BCRABL tyrosine kinase activity, substrates can no longer be phosphorylated and the cellular events leading to unregulated proliferation are interrupted. Adapted, with permission, from Mauro & Druker (2001).

Once rapidly fatal, with a 5-year survival rate of only 20% (NCI, 2012), CML can now be managed as a chronic condition for many patients. This dramatic change in prognosis was made possible by highly effective tyrosine kinase inhibitor (TKI) therapy, which targets the BCR-ABL protein kinase. Imatinib (Gleevec), the first TKI approved by the US Food and Drug Administration (FDA) for CML, has now been followed by the approval of two newer and more potent agents: dasatinib (Sprycel) and nilotinib (Tasigna).

Prior to the advent of TKIs, treatment responses were assessed using hematologic and cytogenetic analyses. Hematologic assessment involves characterization of the cell types in a blood sample; cytogenetic assessment involves microscopic evaluation of chromosomes to quantify the percentage of Philadelphia-positive (Ph+) metaphases. Due to the greater efficacy of TKIs, the number of leukemic cells in the bone marrow decreases to a level that cannot be detected by conventional cytogenetic techniques (Branford, Hughes, & Rudzki, 1999). Therefore, a more sensitive test for monitoring the course of treatment and further quantifying minimal residual disease (MRD) was needed.

The groundbreaking work that led to the identification of the BCR-ABL TK protein as the driver of CML (Daley, Van Etten, & Baltimore, 1990) made it possible to develop a polymerase chain reaction (PCR) assay that measures the amount of *BCR-ABL* messenger RNA (mRNA) in blood cells. Polymerase chain reaction is far more sensitive than hematologic and cytogenetic assessments and can detect the presence of the small number of leukemic cells remaining in patients with MRD (Baccarani, Castagnetti, Gugliotta, Palandri, & Soverini, 2009a; Kantarjian, Schiffer, Jones, & Cortes, 2008). Minimal residual disease can be a source of relapse; therefore, appropriate disease monitoring can have a profound impact on the ultimate course of disease in individual patients (Baccarani et al., 2009a). Increases in BCR-ABL transcript numbers may predict impending loss of response or indicate emergence of a *BCR-ABL* mutation (Jabbour, Cortes, & Kantarjian, 2008). With ongoing, highly sensitive molecular monitoring, early treatment corrections can be made, if necessary, that can optimize responses and increase the probability of long-term survival. As treatment has become more effective, the elimination of BCR-ABL to undetectable levels has become the goal of treatment.

Advanced practitioners (APs) can play an instrumental role in guiding patients through the complexities of molecular testing. Familiarity with treatment milestones (summarized in Table 1) and guidelines for measuring treatment responses prepares APs to communicate the results of monitoring to their patients; this can encourage patients to become actively involved in their own management plans.

**Table 1:**
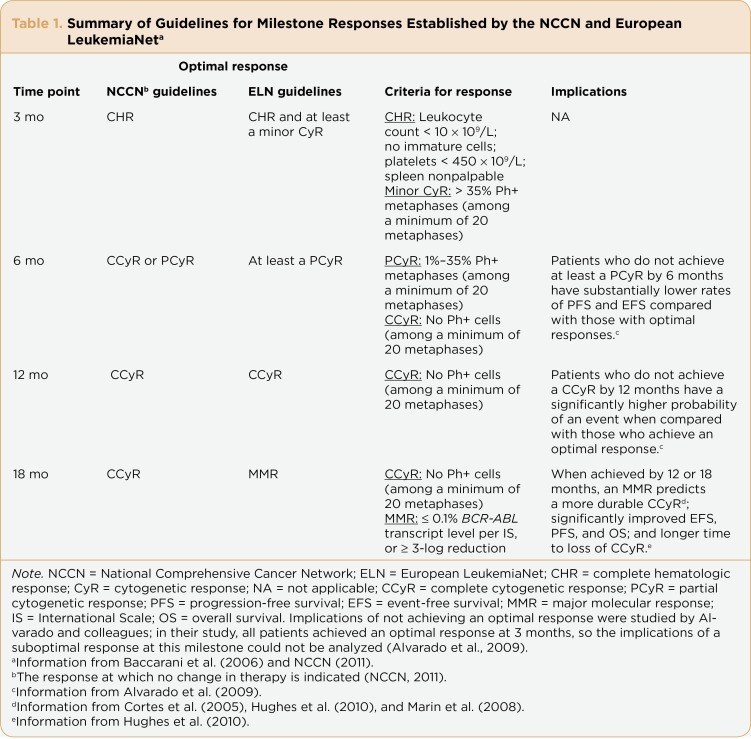
Table 1. Summary of Guidelines for Milestone Responses Established by the NCCN and European LeukemiaNet.

## Importance of Molecular Monitoring

According to the first randomized, phase III study of imatinib—the International Randomized Study of Interferon versus STI571 (IRIS) trial—molecular response was found to provide information regarding the depth and stability of treatment response (Hughes et al., 2003). The investigators analyzed molecular responses in patients who had achieved complete cytogenetic response (CCyR) to determine the ability of TKIs to further reduce disease burden. Patients with at least a 3-log reduction in *BCR-ABL* transcript level had minimal risk of transformation to advanced phases of disease (100% probability of remaining progression-free at 24 months). This level of response was defined as a major molecular response (MMR).

The Evaluating Nilotinib Efficacy and Safety in Clinical Trials—Newly Diagnosed Patients (ENESTnd) trial (Saglio et al., 2010) assessed MMR as the primary endpoint and the DASISION (Dasatinib Versus Imatinib Study in Treatment-Naïve CML Patients) trial (Kantarjian et al., 2010) measured MMR as a secondary endpoint. More patients who received nilotinib or dasatinib achieved MMR at all time points assessed than did patients receiving imatinib (Kantarjian et al., 2010; Saglio et al., 2010). More recent data have extended these findings to 18 and 24 months of follow-up for dasatinib and nilotinib, respectively (Kantarjian et al., 2011; Shah et al., 2010).

As data continue to emerge, it is becoming evident that attaining MMR has important prognostic implications. In the IRIS trial, patients with CCyR who had achieved MMR by 12 and 18 months were less likely to lose CCyR than were patients who had not reached this milestone (Druker et al., 2006; Hughes et al., 2010). The molecular response also appears able to predict long-term outcomes such as overall survival (OS) and progression-free survival (PFS). In a retrospective analysis, patients who achieved stable MMR had increased OS and PFS compared with patients who never achieved MMR (Palandri et al., 2009). In the IRIS study, patients who achieved MMR by 18 months did not progress to advanced disease (accelerated phase or blast crisis) and had a 95% rate of event-free survival at 7 years (Hughes et al., 2010). The authors concluded that achieving MMR sooner was associated with improved long-term outcomes (Hughes et al., 2010). This association between *BCR-ABL* transcript reduction and long-term outcomes supports the use of molecular monitoring to measure response over time (Hughes et al., 2006). Results with front-line nilotinib and dasatinib were consistent with those achieved with imatinib: progression is unlikely once MMR is achieved (Saglio et al., 2010; Kantarjian et al., 2010).

## A PCR Primer

**Figure 2:**
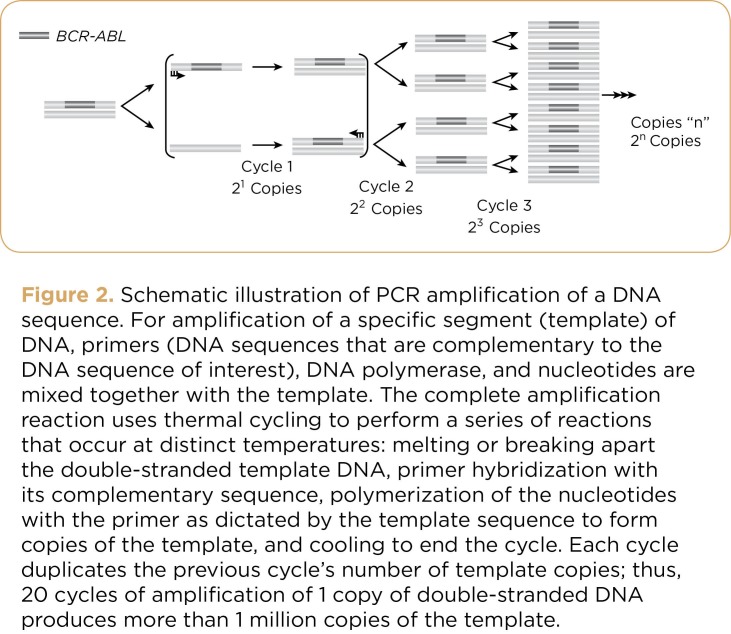
Figure 2. Schematic illustration of PCR amplification of a DNA sequence. For amplification of a specific segment (template) of DNA, primers (DNA sequences that are complementary to the DNA sequence of interest), DNA polymerase, and nucleotides are mixed together with the template. The complete amplification reaction uses thermal cycling to perform a series of reactions that occur at distinct temperatures: melting or breaking apart the double-stranded template DNA, primer hybridization with its complementary sequence, polymerization of the nucleotides with the primer as dictated by the template sequence to form copies of the template, and cooling to end the cycle. Each cycle duplicates the previous cycle’s number of template copies; thus, 20 cycles of amplification of 1 copy of double-stranded DNA produces more than 1 million copies of the template.

Developed in 1983, PCR technology is a simple, readily available technique that makes repeated copies of a piece of DNA of interest. Because the number of copies increases exponentially in PCR, billions of DNA copies can be made in just a few hours, making sufficient quantities of the specific DNA for it to be measured (Figure 2). Reverse transcriptase PCR (RT-PCR) is similar to PCR. Instead of copying DNA, however, the first step of the reaction is to copy the RNA transcript into DNA and then to make copies of the DNA. RT-PCR roughly reflects how much BCR-ABL protein is being made (Hunt, 2010).

Real-time quantitative RT-PCR (qRT-PCR), an enhancement of the RT-PCR technique, enables quantification of mRNA (Branford et al., 1999). In addition to having greater sensitivity than cytogenetic analysis—qRT-PCR can detect 1 CML cell in >= 100,000 normal cells—the strong correlation between results obtained in the bone marrow and peripheral blood permits evaluation of *BCR-ABL* mRNA in either type of sample (Branford et al., 1999). Blood sampling is often chosen for long-term monitoring because it is less invasive, more convenient, and less costly to perform than bone marrow sampling.

A consensus panel convened by the National Institutes of Health in 2005 recommended that qRT-PCR be used in the initial workup of a CML patient to measure *BCR-ABL* transcript numbers before initiation of treatment, to monitor the response to treatment, and to detect MRD (Hughes et al., 2006). It is important to differentiate *qualitative* PCR from *quantitative* PCR. Qualitative PCR detects only the presence or absence of *BCR-ABL* transcript. Qualitative PCR may be useful for diagnosis, but it cannot be used to measure response to treatment (Hughes et al., 2006). Errors in selecting the correct PCR test can be made in the clinic when completing the order form or in the laboratory when performing the PCR test; therefore, it is important to confirm that *quantitative* PCR methods have been ordered and performed.

## Molecular Testing: Practical Aspects

**Figure 3:**
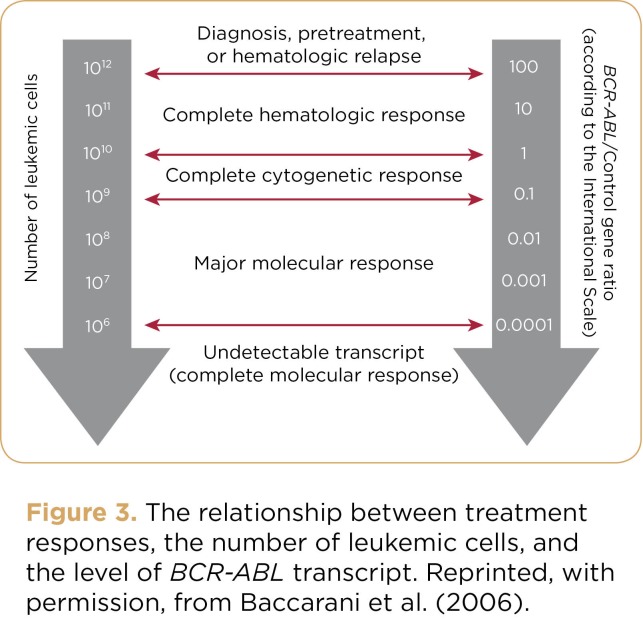
Figure 3. The relationship between treatment responses, the number of leukemic cells, and the level of BCR-ABL transcript. Reprinted, with permission, from Baccarani et al. (2006).

A baseline level of *BCR-ABL* transcript should be obtained from bone marrow before treatment to monitor response to TKI therapy. Thereafter, molecular monitoring by peripheral blood is recommended at 3-month intervals until patients achieve CCyR (recommended by the National Comprehensive Cancer Network [NCCN]) or MMR (recommended by the European LeukemiaNet [ELN] and European Society for Medical Oncology [Baccarani et al., 2009b; Baccarani & Dreyling, 2010]) and at 3- to 6-month intervals thereafter (Baccarani et al., 2009a; Hughes et al., 2006; NCCN, 2011). Figure 3 depicts the relationship between molecular monitoring results and treatment response.

When molecular testing is performed using a peripheral blood sample, 10 mL should be collected by venipuncture (Hughes, 2006) and stored at room temperature. The sample should not be frozen. It is advisable to draw and process blood samples early in the week, ideally within a 24-hour period (and not beyond 36 hours), to avoid degradation of mRNA transcripts over the weekend, when most laboratories are not open. qRT-PCR conducted immediately after blood is drawn provides more accurate *BCR-ABL* values than if the test is performed 24 to 48 hours later, during which time the mRNA sample may have degraded by as much as 50% (Branford et al., 2006). Samples from individual patients should be sent to the same laboratory to ensure that each one is tested under the same conditions. Results received from the same laboratory are likely to reflect actual changes within a patient over time instead of artificial variations that may occur because of different testing methods and procedures used by one laboratory service or another. It is typically 1 week from the time the sample is obtained to when the clinician receives the result.

## Standardization of qRT-PCR Results: The International Scale

An international effort to standardize qRT-PCR results is ongoing. Standardization is important so that results can be compared between laboratories, even when there are differences in methods and procedures. Standardization also provides clinics that treat few CML patients a point of comparison to the published literature. Finally, for multicenter clinical trials, standardization enables investigators to use common clinical values for clinical decisions, compare values obtained from different clinic settings, and consistently interpret clinical research data (Branford et al., 2008).

The International Scale (IS) has been developed to provide a common approach for reporting the results of qRT-PCR. The IS is anchored to two values: (1) a standardized baseline value of 100% and (2) a standardized MMR value set at 0.1%, that is, a 3-log reduction from the standardized baseline (Hughes et al., 2006; Hughes et al., 2003). As a comparison for what a 3-log reduction represents, a CCyR corresponds to approximately a 1- to 2-log reduction in the level of *BCR-ABL* transcripts (10% to 1% on the IS). Although there have been challenges in reconciling qRT-PCR results during the transition to IS (some laboratories have adopted the recommendations and others have not), work in this arena to improve standardization and interpretation of molecular results is ongoing (Hughes et al., 2006; Muller et al., 2009). Responses beyond MMR are also being explored. Although there is no agreed-upon definition, a complete molecular response (CMR) is currently referred to as undetectable *BCR-ABL* transcript levels, generally considered to be > 4.5-log reduction below the standardized baseline on the IS.

The advanced practitioner is a valuable resource in helping patients understand variations in qRT-PCR results when a different laboratory service is used, as illustrated by this vignette:

A patient with CML had been successfully treated with a TKI for several years, along with routine qRT-PCR monitoring. His health insurance company began using an alternate commercial laboratory service several months ago. The previous laboratory used the IS, as used in some laboratory services in the United States and Europe. The new commercial laboratory service, however, uses its own laboratory-specific standard. The current test result shows a higher number of *BCR-ABL* transcripts compared to the last test performed by the previous laboratory. The clinician must now determine the underlying cause of the higher *BCR-ABL* transcript level. The current qRT-PCR result could be interpreted in a number of ways: (1) The patient is beginning to lose response to TKI therapy, (2) the patient is not adhering to the medication regimen, or (3) differences in laboratory standards and procedures yield higher results than the previous laboratory. In this case, the clinician was aware of the possibility of differing laboratory standards and reinterpreted the result using the first laboratory’s standard scale. The standardized value was now consistent with previously attained values, indicating that the patient was still in remission and no change in management was needed.

**Figure 4:**
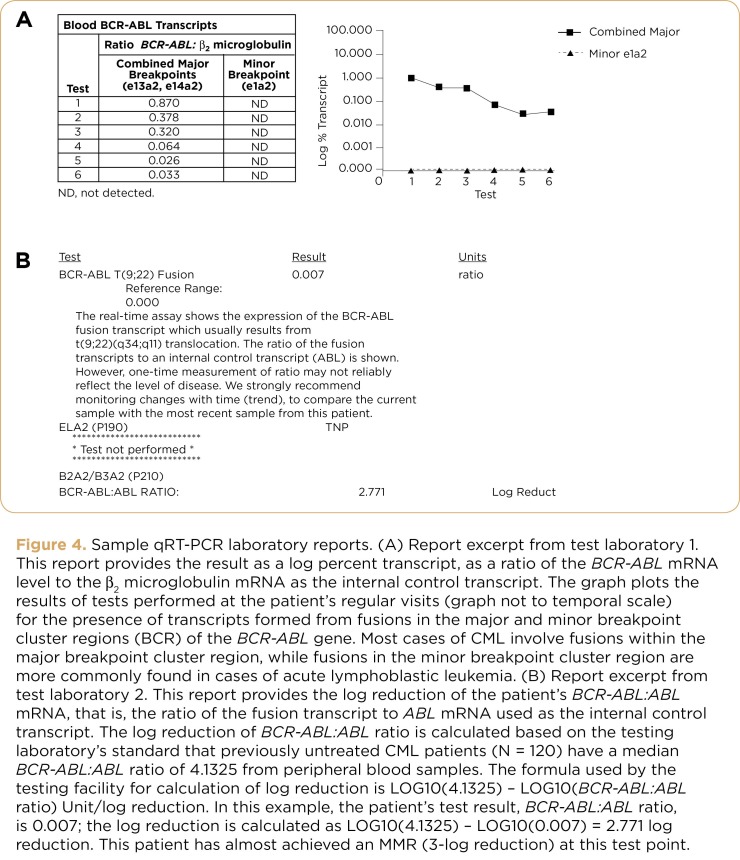
Figure 4. Sample qRT-PCR laboratory reports. (A) Report excerpt from test laboratory 1. This report provides the result as a log percent transcript, as a ratio of the BCR-ABL mRNA level to the β_2_ microglobulin mRNA as the internal control transcript. The graph plots the results of tests performed at the patient’s regular visits (graph not to temporal scale) for the presence of transcripts formed from fusions in the major and minor breakpoint cluster regions (BCR) of the BCR-ABL gene. Most cases of CML involve fusions within the major breakpoint cluster region, while fusions in the minor breakpoint cluster region are more commonly found in cases of acute lymphoblastic leukemia. (B) Report excerpt from test laboratory 2. This report provides the log reduction of the patient’s BCR-ABL:ABL mRNA, that is, the ratio of the fusion transcript to ABL mRNA used as the internal control transcript. The log reduction of BCR-ABL:ABL ratio is calculated based on the testing laboratory’s standard that previously untreated CML patients (N = 120) have a median BCR-ABL:ABL ratio of 4.1325 from peripheral blood samples. The formula used by the testing facility for calculation of log reduction is LOG10(4.1325) – LOG10(BCR-ABL:ABL ratio) Unit/log reduction. In this example, the patient’s test result, BCR-ABL:ABL ratio, is 0.007; the log reduction is calculated as LOG10(4.1325) – LOG10(0.007) = 2.771 log reduction. This patient has almost achieved an MMR (3-log reduction) at this test point.

This case illustrates how important it is to be aware that qRT-PCR results can be reported differently depending on the laboratory. Some laboratories report PCR results in nonpercentage, scientific notation format, and others simply report a raw PCR number without reference points. An example of variable results from two different laboratories testing one patient is shown in Figure 4. In addition, the reference standards differed between the laboratories and units of the results varied. Laboratory 1 presented the data as the ratio of *BCR-ABL* to beta_2_ microglobulin mRNA; laboratory 2 presented the data as the log reduction from their standard value of mean CML patient *BCR-ABL* levels prior to commencement of treatment, without providing an internal reference standard.

In summary, when a patient’s most recent qRT-PCR result differs markedly from previous serial measurements, it is important to explore the reasons behind the change in level. A change in laboratory service could be one such reason. By communicating these issues with patients, the AP can reassure them that their increased *BCR-ABL* levels do not necessarily mean their response has been lost.

## Engaging Patients in Monitoring Their Molecular Response

**Figure 5:**
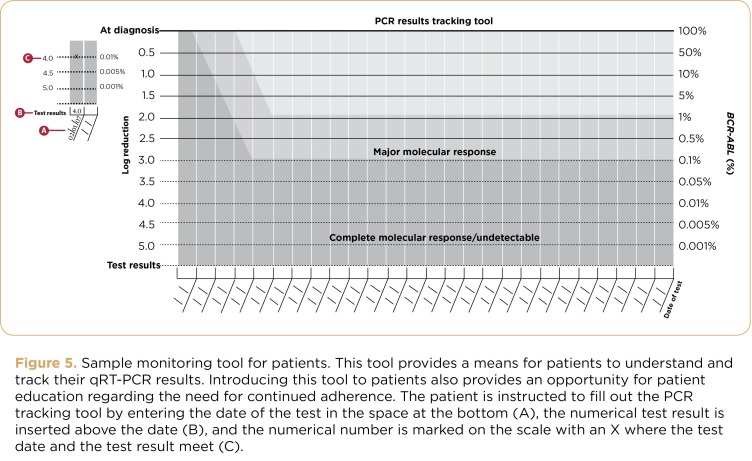
Figure 5. Sample monitoring tool for patients. This tool provides a means for patients to understand and track their qRT-PCR results. Introducing this tool to patients also provides an opportunity for patient education regarding the need for continued adherence. The patient is instructed to fill out the PCR tracking tool by entering the date of the test in the space at the bottom (A), the numerical test result is inserted above the date (B), and the numerical number is marked on the scale with an X where the test date and the test result meet (C).

Translating molecular testing results for patients helps them to understand their response and whether treatment will be continued or a change in treatment will be considered. CML is not cured by TKI therapy; therefore, patients need to receive lifelong treatment. A recent study indicated that optimal responses are achieved in patients who are adherent to TKI therapy (Marin et al., 2010). Understanding of and participation in monitoring is a powerful reminder of the need for continued therapy. Fostering active participation in patients’ treatment plans using tracking tools (Figure 5) can ensure that patients are adhering well to therapy.

Advanced practitioners can assist patients in interpreting numbers over time and avoiding focusing on the results of a single test, because longer-term PCR trends are more important than any individual test result. *BCR-ABL* transcript numbers will follow one of four patterns: declining transcript levels, undetectable transcript levels, stable (or at a plateau) transcript levels, or increasing transcript levels (Hughes et al., 2006). Patients should be evaluated for and educated as to the possible reasons for a rising *BCR-ABL* level: nonadherence, drug-drug or drug-food interactions, assay error, different laboratory or different laboratory procedure, or impending relapse. Molecular monitoring results can be used to educate patients, similar to the use of monitoring blood pressure in patients with hypertension or glucose readings in patients with diabetes. These results reflect response to therapy and may signal when a change in treatment is needed. Showing the patient’s BCR-ABL transcript pattern over time creates a visual image that may help patients understand their response and management approach.

Patients should know that an increase in the BCR-ABL transcript number is not an immediate cause for concern. As illustrated in the patient vignette, the sample could have been sent to a different laboratory or the laboratory could have altered its protocol. The test should be repeated to determine the cause and potential need for change in treatment. If a trend of rising *BCR-ABL* transcripts emerges, a change in treatment strategy is normally warranted.

## Conclusions

Molecular monitoring is the most sensitive tool available for tracking response to treatment and predicting outcomes (Radich, 2009). The NCCN and ELN guidelines recommend molecular monitoring as an important aspect of patient management (Baccarani et al., 2009b; NCCN, 2011). In turn, it is critically important that the test is accurate. The IS is an important step toward standardizing molecular monitoring results (Hughes et al., 2006).

For APs, it is important to bear in mind that a patient’s outlook on the latest *BCR-ABL* level can affect how they react to their prognosis. Advanced practitioners can educate their patients so that they may understand the results of their tests. Undetectable *BCR-ABL* levels can bring feelings of relief, whereas an increasing level can lead to anxiety. Advanced practitioners therefore serve as a valuable resource for interpreting results for patients, answering questions, encouraging continued adherence to treatment, and alleviating concerns.

Molecular monitoring represents one of the most recent advances in patient management. The field of CML has evolved relatively rapidly over the past decade. With the increased potency of therapies and the improved sensitivity of monitoring techniques, clinicians and patients have additional tools to more effectively manage CML over the long term.

## Acknowledgments

Financial support for medical editorial assistance was provided by Novartis Pharmaceuticals. We thank David Keleti, PhD, Patricia Segarini, PhD, and Mariana Ovnic, PhD, for their medical editorial assistance.
